# Comparison between high-flow nasal oxygen (HFNO) alternated with non-invasive ventilation (NIV) and HFNO and NIV alone in patients with COVID-19: a retrospective cohort study

**DOI:** 10.1186/s40001-024-01826-3

**Published:** 2024-04-22

**Authors:** Amanda Pereira da Cruz, Gloria Martins, Camila Marinelli Martins, Victoria Marques, Samantha Christovam, Denise Battaglini, Chiara Robba, Paolo Pelosi, Patricia Rieken Macedo Rocco, Fernanda Ferreira Cruz, Cynthia dos Santos Samary, Pedro Leme Silva

**Affiliations:** 1 Laboratory of Pulmonary Investigation, Institute of Biophysics Carlos Chagas Filho, Centro de Ciências da Saúde, Federal University of Rio de Janeiro, Avenida Carlos Chagas Filho, 273, Bloco G-014, Ilha do Fundão, Rio de Janeiro, RJ 21941-902 Brazil; 2 D’or Institute of Research and Teaching, Barra D’or Hospital, Rio de Janeiro, Brazil; 3 AAC&T Research Consulting LTDA, Curitiba, Brazil; 4 Department of Cardiorespiratory and Musculoskeletal Physiotherapy, Faculty of Physiotherapy, Federal University of Rio de Janeiro, Rio de Janeiro, Brazil; 5 IRCCS Ospedale Policlinico San Martino, Genoa, Italy; 6 Department of Surgical Sciences and Integrated Diagnostics (DISC), University of Genoa, Genoa, Italy

**Keywords:** COVID-19, Non-invasive ventilation, High-flow nasal oxygen, Oxygen therapy, Invasive mechanical ventilation

## Abstract

**Background:**

Non-invasive respiratory support (conventional oxygen therapy [COT], non-invasive ventilation [NIV], high-flow nasal oxygen [HFNO], and NIV alternated with HFNO [NIV + HFNO] may reduce the need for invasive mechanical ventilation (IMV) in patients with COVID-19. The outcome of patients treated non-invasively depends on clinical severity at admission. We assessed the need for IMV according to NIV, HFNO, and NIV + HFNO in patients with COVID-19 according to disease severity and evaluated in-hospital survival rates and hospital and intensive care unit (ICU) lengths of stay.

**Methods:**

This cohort study was conducted using data collected between March 2020 and July 2021. Patients ≥ 18 years admitted to the ICU with a diagnosis of COVID-19 were included. Patients hospitalized for < 3 days, receiving therapy (COT, NIV, HFNO, or NIV + HFNO) for < 48 h, pregnant, and with no primary outcome data were excluded. The COT group was used as reference for multivariate Cox regression model adjustment.

**Results:**

Of 1371 patients screened, 958 were eligible: 692 (72.2%) on COT, 92 (9.6%) on NIV, 31 (3.2%) on HFNO, and 143 (14.9%) on NIV + HFNO. The results for the patients in each group were as follows: median age (interquartile range): NIV (64 [49–79] years), HFNO (62 [55–70] years), NIV + HFNO (62 [48–72] years) (*p* = 0.615); heart failure: NIV (54.5%), HFNO (36.3%), NIV + HFNO (9%) (*p* = 0.003); diabetes mellitus: HFNO (17.6%), NIV + HFNO (44.7%) (*p* = 0.048). > 50% lung damage on chest computed tomography (CT): NIV (13.3%), HFNO (15%), NIV + HFNO (71.6%) (*p* = 0.038); SpO_2_/FiO_2_: NIV (271 [118–365] mmHg), HFNO (317 [254–420] mmHg), NIV + HFNO (229 [102–317] mmHg) (*p* = 0.001); rate of IMV: NIV (26.1%, p = 0.002), HFNO (22.6%, p = 0.023), NIV + HFNO (46.8%); survival rate: HFNO (83.9%), NIV + HFNO (63.6%) (*p* = 0.027); ICU length of stay: NIV (8.5 [5–14] days), NIV + HFNO (15 [10–25] days (*p* < 0.001); hospital length of stay: NIV (13 [10–21] days), NIV + HFNO (20 [15–30] days) (*p* < 0.001). After adjusting for comorbidities, chest CT score and SpO_2_/FiO_2_, the risk of IMV in patients on NIV + HFNO remained high (hazard ratio, 1.88; 95% confidence interval, 1.17–3.04).

**Conclusions:**

In patients with COVID-19, NIV alternating with HFNO was associated with a higher rate of IMV independent of the presence of comorbidities, chest CT score and SpO_2_/FiO_2_.

*Trial registration* ClinicalTrials.gov identifier: NCT05579080.

**Supplementary Information:**

The online version contains supplementary material available at 10.1186/s40001-024-01826-3.

## Introduction

COVID-19 is caused by the SARS-CoV-2 virus and leads to an exacerbated inflammatory response in the host [1]. Non-invasive respiratory support (conventional oxygen therapy [COT], non-invasive ventilation [NIV], high-flow nasal oxygen [HFNO], as well as NIV alternating with HFNO [NIV + HFNO] [2, 3] are potential therapeutic strategies to prevent intubation in patients with COVID-19. However, different methods of non-invasive respiratory support may result in different outcomes, which may be associated with the baseline clinical characteristics of patients with COVID-19 [4–8] and the extent of lung damage on chest computed tomography (CT) images [9]. In addition, patients with COVID-19 present up to a twofold higher risk of failure of non-invasive respiratory support compared with patients who do not have COVID-19 when HFNO or NIV is used as first-choice ventilatory therapy [10]. NIV increases arterial oxygenation by reducing alveolar collapse and improving ventilation–perfusion matching [11]. HFNO has been used in patients with COVID-19 as an initial strategy to reduce anatomic dead space, respiratory rate, and inspiratory effort, and improve respiratory mechanics and end-expiratory lung volume, thus preventing the progression of lung injury [12]. Both NIV and HFNO can help avoid the complications associated with IMV, such as ventilator-induced lung injury, cardiovascular impairment, and infectious diseases [13]. Nevertheless, a long period of vigorous ventilatory effort during NIV or HFNO may worsen lung damage by several mechanisms gathered under the name “patient self-inflicted lung injury” (P-SILI), thus increasing the risk of intubation [14–16]. The use of NIV + HFNO could limit prolonged NIV sessions and thus P-SILI, without marked impairment of oxygenation between NIV sessions and with a relatively low intubation rate [17]. In addition, adjustments to the baseline conditions of patients with COVID-19 are required to properly compare NIV, HFNO, and NIV + HFNO in terms of clinical outcomes. The primary aim of this retrospective single-center study was to evaluate if NIV + HFNO increased the risk of IMV in patients with COVID-19 compared with HFNO and NIV alone. Secondary aims included the in-hospital mortality rate, and hospital and intensive care unit (ICU) lengths of stay for different non-invasive respiratory support strategies.

## Materials and methods

### Study design

This single-center, retrospective cohort study evaluated patients admitted to the ICU of Hospital Barra D'or between March 2020 and July 2021. The study protocol was approved by the Co-substantiated Ethics and Research Committee of the Research and Teaching Institute D’or, on 3 December 2021 (CAAE: 52534221.5.0000.5249). This study was registered in the Brazilian Registry of Clinical Trials (REBEC; number RBR-3vs3gh8) and ClinicalTrials.gov (NCT05579080). This report follows the Strengthening the Reporting of Observational Studies in Epidemiology (STROBE): Explanation and Elaboration guidelines [18].

### Patient eligibility

The inclusion criteria for the patients were as follows: > 18 years of age; admitted to the hospital for COVID-19 confirmed by a positive result of a real-time reverse transcriptase–polymerase chain reaction assay from a nasal or pharyngeal swab or chest CT suggestive of pneumonia caused by COVID-19 who needed COT, NIV, HFNO, or NIV + HFNO. The exclusion criteria were hospital length of stay < 3 days; patients who evolved to endotracheal intubation with IMV within 48 h of hospitalization; patients on NIV and/or HFNO for < 48 h because they may need IMV for reasons not directly related to non-invasive respiratory support; patients with medical records lacking outcome variables, patients hospitalized for other causes, absence of consent, and pregnancy; and patients who received COT, NIV or HFNO after extubation.

### Data collection

The following data were collected at hospital admission: demographic data (age, gender, weight, height, body mass index [BMI]), comorbidities (chronic respiratory disease, chronic obstructive pulmonary disease, asthma, diabetes mellitus, systemic arterial hypertension, cerebrovascular disease, heart disease, structural lung disease, cerebrovascular disease, heart failure, arterial hypertension, kidney failure, immunosuppression, dementia), time from symptom onset to hospital admission, and chest CT with data on the proportion of lung involvement. Briefly, the major CT findings were described using international standard nomenclature defined by the Fleischner Society glossary and peer-reviewed literature on viral pneumonia, using terms, including ground-glass opacity, crazy-paving pattern, pleural effusion, and consolidation [19, 20]. Data on the peripheral oxygen saturation (SpO_2_) and inspired oxygen fraction (FiO_2_) ratio, respiratory rate (RR) and respiratory rate–oxygentation (ROX) index, the use of medication during the study (azithromycin, amoxicillin/clavulanic acid, or dexamethasone), renal replacement therapy, and prognostic score (SAPS-3) were also collected. Information on the duration of ventilatory support (NIV, HFNO, and NIV + HFNO), inspiratory and expiratory airway pressures during NIV and NIV + HFNO used between ventilatory supports, and highest and lowest oxygen flow during HFNO was also obtained. For all groups, details of hospital and ICU lengths of stay, as well as the time to invasive mechanical ventilation and in-hospital mortality rate were obtained from electronic medical records. All data were collected using the WPD hospital informatics system.

### Definitions

Clinical decisions were made according to hospital protocol, which followed the international recommendations for clinical management of COVID-19 and the Brazilian recommendations from the Federal Health Agency [21].

At hospital admission, patients with clinical symptoms and signs of acute respiratory failure with a diagnosis of COVID-19 were allocated to a suitable facility. If the patient presented with hypoxemia (partial pressure of oxygen [PaO_2_] ≤ 60 mmHg or peripheral O_2_ saturation [SpO_2_] ≤ 88%), supplemental oxygen was started immediately at between 1 and 15 L/min (nasal cannula [1–6], oxygen face mask [7–9 L/min], or reservoir mask [10–15 L/min]). If work of breathing and dyspnea were detected in the absence of a need for emergency endotracheal intubation (characterized by a lowered level of consciousness; Glasgow Coma Scale score < 8, SpO_2_ < 88%, intense respiratory effort with the use of accessory muscles, pneumothorax not drained, and cardiac arrest), the patient was started on NIV (in cases of predominance of respiratory distress) through an interface (oronasal or full face mask) or HFNO (in cases of predominance of hypoxemia with PaO_2_ < 60 mmHg). The minimum exposure to therapy was at least 48 h for all groups, and at least 180 min/day (continuously or 2–3 times per day) in the NIV group or 24 h/day in the HFNO group. If the patient presented with persistent hypoxemia (SpO_2_ < 93% with supplementary oxygen until 15 L/min in a reservoir mask) during the NIV intervals, HFNO was applied (Additional file 1: Fig. S1).

NIV through a Puritan Bennet 840 ventilator was first applied continuously. based on clinical improvement, NIV was reduced to 2–3 times per day, up to 240 min each session. Time under NIV was reduced progressively until weaning. Supplementary oxygen therapy was given after NIV if necessary to maintain SpO_2_ > 90% via a nasal catheter (1–5 L/min), a simple oxygen mask (6–10 L/min), or a reservoir mask (6–15 L/min). HFNO (Vapotherm or Optiflow device according to availability) was used according to the level of flow and oxygen inspired fraction and was reduced progressively until weaning.

Failure of non-invasive respiratory support was defined as the need for endotracheal intubation with IMV according to the following criteria: clinical decision by the medical team; hypoxemia (PaO_2_ ≤ 60 mmHg) and/or acidosis (pH ≤ 7.35); low level of consciousness; worsening of the work of breathing; cardiopulmonary resuscitation event; intolerance to therapy and/or face mask, or other [21].

### Outcomes

The primary outcome was the need for IMV in those patients with COVID-19 who had previously undergone NIV, HFNO, or NIV + HFNO. The secondary aims were to describe the in-hospital mortality rate and hospital and ICU lengths of stay in the same population and subgroups.

### Statistical analysis

There was no sample size calculation due to the exploratory, descriptive, and retrospective nature of this study. All cases from March 2020 to July 2021 were considered eligible as long as they met the inclusion criteria and did not meet the exclusion criteria. For descriptive summary statistics, variables are reported as means (standard deviation), medians (interquartile range, 25–75%), or absolute and relative frequencies, as appropriate. Pairwise comparisons using Wilcoxon’s rank sum test with continuity correction and Bonferroni multiple comparison tests were done for all groups according to proportions or continuous variables, as appropriate. IMV and in-hospital mortality rates were analyzed using Kaplan–Meier estimates, and the log-rank test was used for comparisons among the groups. The Kruskal–Wallis test followed by Dunn’s test was applied to assess differences in hospital, ICU lengths of stay and time to invasive mechanical ventilation. The multivariate Cox regression model was applied to adjust the area of impairment on chest CT images to the proportion of lung involvement, the presence of at least one comorbidity and SpO_2_/FiO_2_ ratio to the IMV rate and in-hospital mortality in the three groups. The COT group was used as reference for hazard ratios (HRs) and multivariate Cox regression model adjustment. HRs are presented along with the respective 95% confidence intervals (CIs). All analyses were considered significant when *p* < 0.05, and the analyses were performed in the R 4.0.4 environment [22].

## Results

### Characteristics of patients with COVID-19

**Fig. 1:**
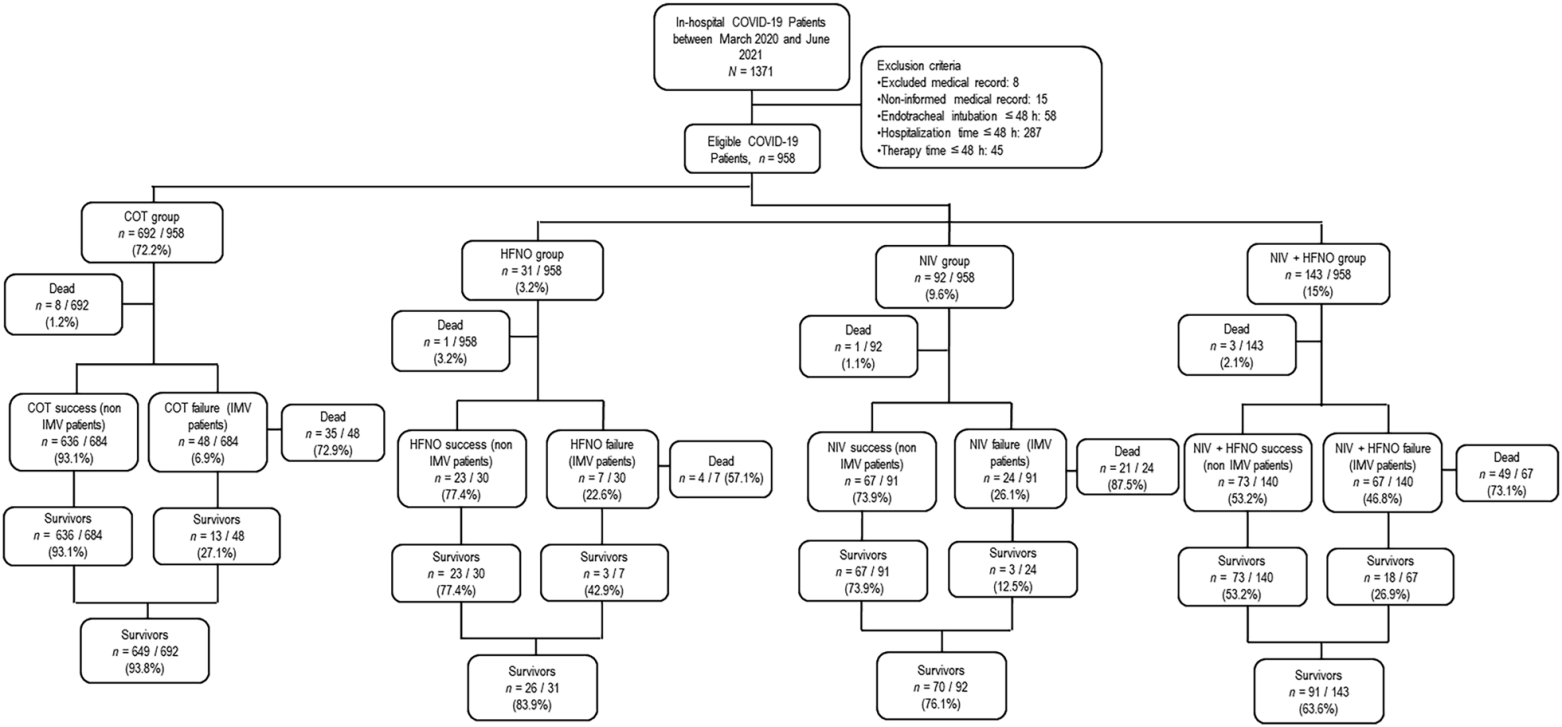
Flowchart of the study. *COT* conventional oxygen therapy, *HFNO* high-flow nasal oxygen, *NIV* non-invasive ventilation. The COT group was used as reference for multivariate Cox regression model adjustment

From March 2020 to July 2021, 1371 patients were screened and 958 were considered eligible (Fig. 1); 692 (72.2%) received COT. COT was treated as the reference group (Additional file 2: Table S1). In addition, 92 (9.6%), 31 (3.2%), and 143 (19.4%) patients received NIV, HFNO, and NIV + HFNO, respectively (Fig. 1). Table 1 depicts the characteristics of patients with COVID-19 admitted to the hospital between March 2020 and July 2021 who underwent NIV, HFNO, or NIV + HFNO. Heart failure was more frequent in the NIV and HFNO groups (54.5% and 36.3%) than the NIV + HFNO group (9%, *p* = 0.003); diabetes mellitus was more prevalent in the NIV + HFNO group (44.7%) than the HFNO group (17.6%, *p* = 0.048). There were more patients with > 50% lung damage on chest CT in the NIV + HFNO group compared with the NIV and HFNO groups (71.6% vs 13.3% and 15%, respectively; *p* = 0.038). SpO_2_/FiO_2_ ratio was lower in NIV + HFNO compared to HFNO groups (229 [102–317] vs 317 [254–420], respectively; *p* = 0.001). RR did not differ among group (*p* = 0.178). ROX index at admission was lower in NIV + HFNO compared to HFNO groups (9.3 [4.9–15.8] vs 16.8 [11–22.4], respectively; *p* = 0.002). Use of azithromycin and amoxicillin/clavulanic acid was higher in the NIV group (35 and 42.1%) and the NIV + HFNO group (48.5 and 31.6%) than in the HFNO group (16 and 26.3%) (*p* = 0.026 and *p* = 0.002, respectively). In addition, the use of dexamethasone was higher in the NIV + HFNO group than in the NIV and HFNO groups (57.6 vs 33.9% and 8.4%, respectively; *p* < 0.001).

**Table 1:** _Characteristics of patients with COVID-19 admitted to the hospital between March 2020 and July 2021_

	All patients	NIV group	HFNO group	NIV + HFNO group	*P* value
Absolute and relative frequencies, *n* (%)	266	92 (34.5)	31 (11.6)	143 (53.8)	
Age (years), median (IQR)	62 (49–73)	64 (49–79)	62 (55–71)	62 (48–72)	0.615
Sex, *n* (%)					
Male	185 (69.5)	59 (31.9)	22 (11.9)	104 (56.2)	0.370
Female	81 (30.4)	33 (40.7)	9 (11.1)	39 (48.1)	
Comorbidities, *n* (%)					
COPD	13 (4.9)	5 (38.4)	1 (7.7)	7 (53.8)	0.885
Asthma	15 (5.6)	5 (33.3)	0 (0)	10 (66.6)	0.308
Heart failure	11 (4.1)	6 (54.5)†	4 (36.3)†	1 (9)	0.003
Arterial hypertension	166 (62.4)	55 (33.1)	20 (12)	91 (54.8)	0.810
Diabetes mellitus	85 (31.9)	32 (37.6)	15 (17.6)	38 (44.7)#	0.048
Kidney failure	21 (7.9)	10 (47.6)	4 (19)	7 (33.3)	0.138
Dementia	8 (3)	7 (87.5)^†#^	0 (0)	1 (12.5)	0.006
Other comorbidities	169 (60.5)	62 (36.7)	16 (9.4)	91 (53.8)	0.463
SAPS-3 score, median (IQR)^a^	48 (43–54)	47 (44–54)	45.5 (42–49)	49 (44–54)	0.076
BMI, *n* (%)					
< 30 kg/m^2^	113 (42.5)	41 (36.2)	12 (10.6)	60 (53.9)	0.446
≥ 30.0 kg/m^2^	91 (34.2)	26 (28.6)	13 (14.2)	52 (57.1)	
Clinical parameters					
Time from symptom onset to hospital admission, *n* (%)					
0–10 days	132 (49.6)	45 (34.1)	16 (12.1)	71 (53.7)	0.052
11–20 days	10 (3.8)	2 (20)	4 (40)	4 (40)	
21– > 30 days	5 (1.5)	1 (20)	2 (40)	2 (40)	
Chest CT score, *n* (%)					
0%–25%	15 (5.6)	6 (40)	1 (6.6)	8 (53.3)	0.038
26%–50%	164 (61.6)	62 (37.8)	19 (11.5)	83 (50.6)	
> 50%	60 (22.6)	8 (13.3)	9 (15)	43 (71.6)^*#^	
Undetermined	3 (1.1)	1 (33.3)	0 (0)	2 (66.6)	
Normal CT scan	24 (9)				
SpO_2_/FiO_2_, median (IQR)	251 [111–356]	271 [118–365]	317 [254 – 420]	229 [102 – 317] ^#^	0.001
RR, median (IQR)	20 [18–25]	20 [19–24]	20 [17–21]	21 [18–26]	0.178
ROX, median (IQR)	11.2 [5.4–18.5]	12.3 [5.2–19.4]	16.8 [11–22.4]	9.3 [4.9–15.8] ^#^	0.002
Concomitant medications, *n* (%)					
Azithromycin	140 (52.6)	49 (35)^#^	23 (16)	68 (48.5)^#^	0.026
Amoxicillin/clavulanic acid	38 (14.3)	16 (42.1)^#^	10 (26.3)	12 (31.6)^#^	0.002
Dexamethasone	236 (88.7)	80 (33.9)^#^	20 (8.4)	136 (57.6)^*#^	< 0.001
Kidney replacement therapy for acute kidney injury (dialysis), *n* (%)	71 (26.7)	22 (31)	5 (7)	44 (62)	0.259

The descriptive analysis of the data is presented as absolute frequencies (*n*) and percentages according to the group except where indicated otherwise. Bonferroni multiple comparison tests were done for proportions or continuous variables, as appropriate

*NIV* non-invasive ventilation *HFNO* high-flow nasal oxygen, *IQR* interquartile range, *COPD* chronic obstructive pulmonary disease, *SAPS* Simplified Acute Physiology Score, *BMI* body mass index (calculated as weight in kilograms divided by height in meters squared), *CT* computed tomography, *SpO*_*2*_*/FiO*_*2*_ peripheral oxygen saturation to inspired oxygen fraction ratio, *RR* respiratory rate, *ROX* Respiratory rate–OXygentation index

^*^Versus the NIV group

^#^Versus the HFNO group

^†^Versus the NIV + HFNO group

^a^SAPS-3 estimates the probability of mortality for patients in the intensive care unit (ICU) on admission using patient characteristics, indication for ICU admission, and physiologic derangement on ICU admission

^b^Data on comorbidities were obtained from the medical records

### IMV, survival rate, and ICU/hospital lengths of stay between the groups

**Fig. 2:**
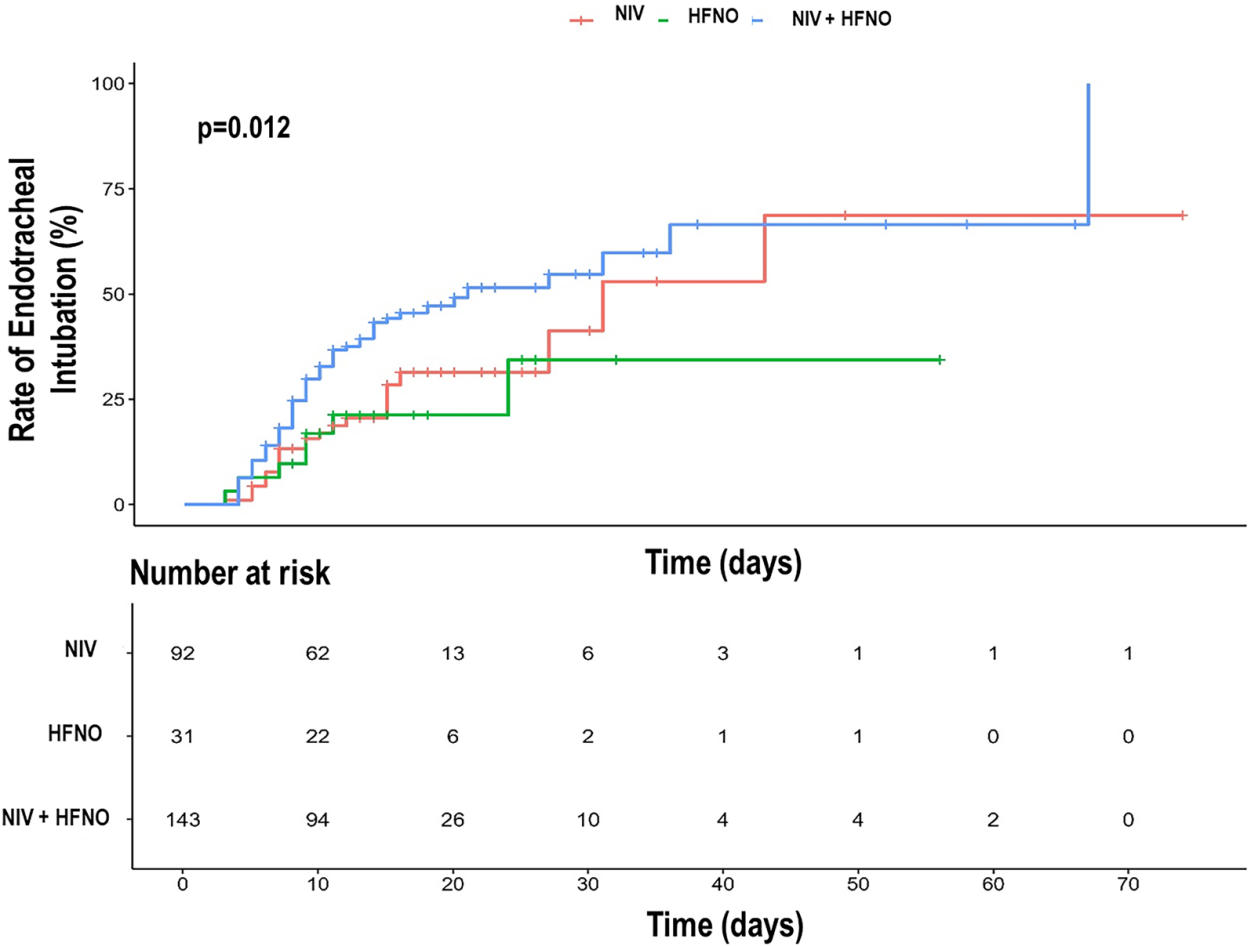
Kaplan–Meier curve of the rate of invasive mechanical ventilation rate according to the groups. *NIV* non-invasive ventilation, *HFNO* high-flow nasal oxygen

**Fig. 3:**
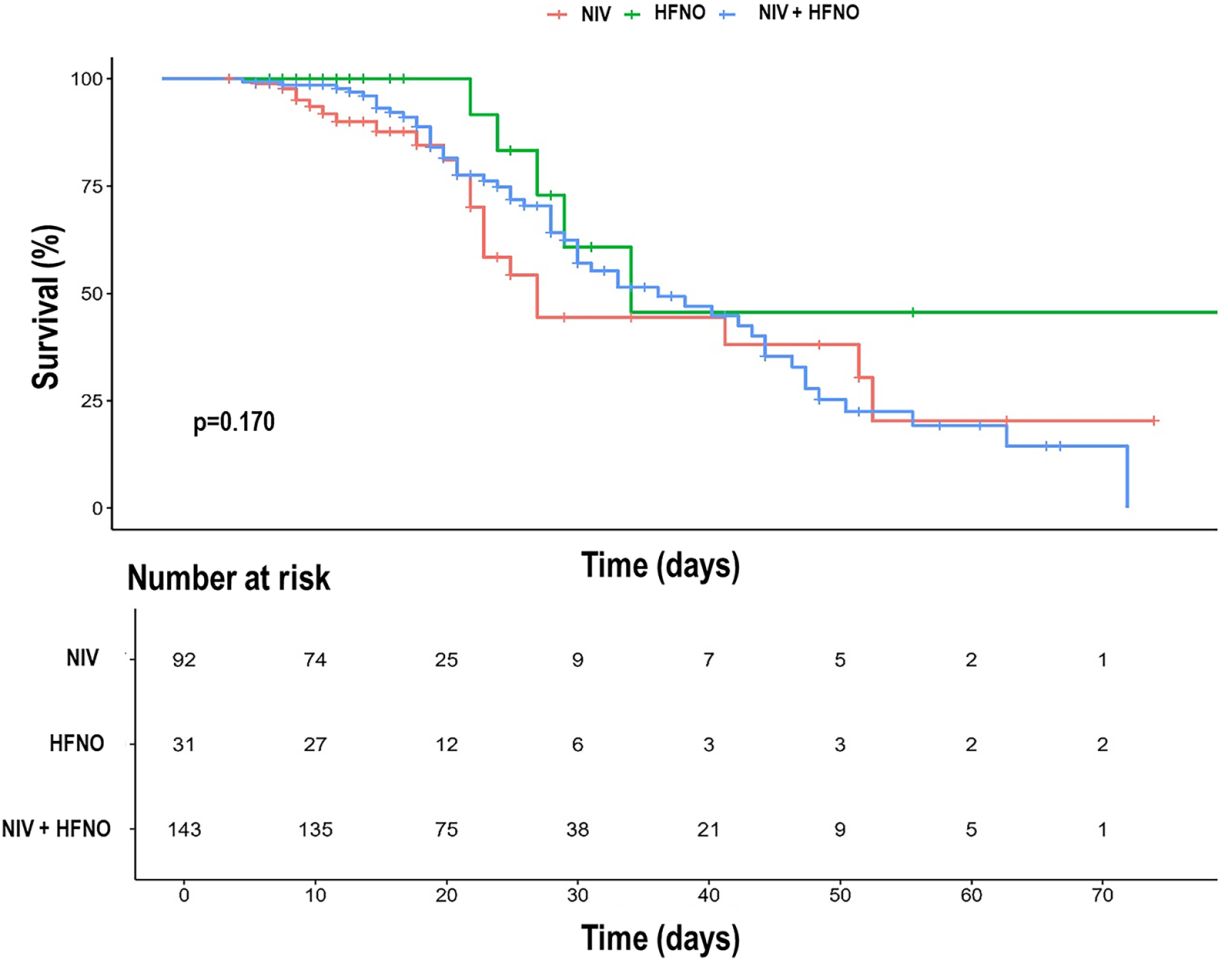
Kaplan–Meier curve of the survival rate according to the groups. *NIV* non-invasive ventilation, *HFNO* high-flow nasal oxygen

The Kaplan–Meier curve of the probability of IMV over time is depicted in Fig. 2 (log-rank *p* = 0.012). The NIV, HFNO, and NIV + HFNO groups showed HRs (95% CIs) of 2.05 (1.25–3.37), 1.64 (0.74–3.65), and 3.60 (2.46–5.27) for the probability of IMV, respectively. The rate of IMV was higher in the NIV + HFNO group than in the NIV and HFNO groups in the population at risk (46.8 vs 26.1% and 22.6%, *p* = 0.002 and *p* = 0.023, respectively). The Kaplan–Meier curve of the survival rate over time is depicted in Fig. 3 (log-rank *p* = 0.170). The NIV, HFNO, and NIV + HFNO groups showed HRs (95% CIs) of 1.82 (1.08–3.07), 0.75 (0.29–1.89), and 1.53 (1.01–2.33) for the survival rate, respectively. The survival rate was lower in the NIV + HFNO group than in the HFNO group (63.6 vs 83.9%, *p* = 0.049). The ICU and hospital lengths of stay were higher in the NIV + HFNO group (15 days [10–25 days] and 20 days [15–30 days]) than the NIV group (8.5 days [5–14 days] and 13 days [10–21 days]; *p* < 0.001 for both) (Table 2). The time to invasive mechanical ventilation did not differ among groups (*p* = 0.837) (Table 2).

**Table 2:** _Secondary outcomes_

	All patients (*n* = 266)	NIV (*n* = 92)	HFNO (*n* = 31)	NIV + HFNO (*n* = 143)	*p* value
ICU length of stay, (days)	7 (3–15)	8.5 (5–14)	9 (7–23)	15 (10–25)*	< 0.001
Hospital length of stay, (days)	8 (5–15)	13 (10–21)	17 (11.5–27)	20 (15–30)*	< 0.001

	All patients who were in IMV (*n* = 98)	NIV (*n* = 24)	HFNO (*n* = 7)	NIV + HFNO (*n* = 67)	*p* value
Time to IMV, (days)	8 (6–12)	8 (6–15)	9 (5.5–10)	8 (6–11)	0.837

Values are medians (interquartile range). Bonferroni multiple comparison tests were done for proportions or continuous variables, as appropriate

*IMV* invasive mechanical ventilation, *NIV* non-invasive ventilation, *HFNO* high-flow nasal oxygen, *ICU* intensive care unit

^*^Versus the NIV group

### Additional analyses of ventilation

The duration of therapy was higher in the NIV + HFNO group (8 days [5–11 days]) than the NIV group (6 days [4–8 days]) and the HFNO group (5 days [4–8 days]; *p* < 0.001) (Additional file 3: Table S2). The inspiratory airway pressure in the NIV and NIV + HFNO groups was adjusted in the range of 8–10 cmH_2_O in 51.9% and 48.9% of patients, respectively. The expiratory airway pressure in the NIV and NIV + HFNO groups was adjusted in the range of 8–9 cmH_2_O in 52.6 and 51.1% of patients, respectively. The oxygen therapy of 2–5 L/min used between ventilatory therapies was higher in the NIV group than the HFNO and NIV + HFNO groups. In addition, more patients in the HFNO group received oxygen therapy of 6–9 L/min compared with the NIV and NIV + HFNO groups (*p* = 0.003). There was no difference in the highest and lowest values for oxygen flow during HFNO and NIV + HFNO (Additional file 3: Table S2). Overall, the major causes of failure of non-invasive therapy in the NIV, HFNO, and NIV + HFNO groups among patients who needed IMV was worsening of the work of breathing (50.7%). Low level of consciousness was higher in the NIV and HFNO groups compared with the NIV + HFNO group (Additional file 4: Table S3).

### Adjusted by the multivariate Cox regression model

When adjusted by chest CT score, the NIV + HFNO group (HR, 3.26; 95% CI 2.12–5.02) was associated with a higher need for IMV. When adjusted by the presence of at least one comorbidity, the NIV (HR, 1.96; 95% CI 1.19–3.22) and NIV + HFNO (HR, 3.44; 95% CI 2.35–5.03) groups were associated with a higher need for IMV. When adjusted by SpO_2_/FiO_2_ ratio, the NIV + HFNO group (HR, 2.07; 95% CI 1.35–3.19) was associated with a higher need for IMV. The NIV + HFNO group showed an association for the rate of IMV, even adjusted for all factors (chest CT score, the presence of at least one comorbidity and SpO_2_/FiO_2_ ratio) (HR, 1.88; 95% CI 1.17–3.04) (Table 3).

**Table 3:** Multivariate Cox model for the rate of invasive mechanical ventilation and in-hospital mortality

	Unadjusted model	Adjusted by CT score	Adjusted by the presence of at least one co-morbidity	Adjusted by SpO_2_/FiO_2_	Adjusted by CT score, presence of at least one co-morbidity and SpO_2_/FiO_2_
	Invasive mechanical ventilation rate				
COT	Reference	Reference	Reference	Reference	Reference
NIV	2.05 (1.25–3.37)	1.37 (0.72–2.59)	1.96 (1.19-3.22)	1.44 (0.85-2.43)	0.97 (0.50–1.88)
HFNO	1.64 (0.74–3.65)	1.71 (0.75–3.88)	1.56 (0.70-3.47)	1.09 (0.49-2.44)	1.08 (0.47–2.47)
NIV+HFNO	3.60 (2.46–5.27)	3.26 (2.12–5.02)	3.44 (2.35-5.03)	2.07 (1.35-3.19)	1.88 (1.17–3.04)
	In-hospital mortality				
COT	Reference	Reference	Reference		Reference
NIV	1.82 (1.08–3.07)	1.29 (0.63–2.63)	1.80 (1.06-3.04)	1.90 (1.10-3.27)	1.25 (0.60–2.59)
HFNO	0.75 (0.29–1.89)	0.68 (0.24–1.93)	0.74 (0.29-1.88)	0.71 (0.28-1.80)	0.59 (0.20–1.70)
NIV+HFNO	1.53 (1.01–2.33)	1.51 (0.94–2.44)	1.54 (1.02-2.34)	1.48 (0.91-2.40)	1.53 (0.88-2.67)

Values are hazard ratios (95% confidence intervals)

*CT* computed tomography, *COT* conventional oxygen therapy, *NIV* non-invasive ventilation, *HFNO* high-flow nasal oxygen

When adjusted by the presence of at least one comorbidity, the NIV (HR, 1.80; 95% CI 1.06–3.04) and NIV + HFNO (HR, 1.54; 95% CI 1.02–2.34) groups were associated with in-hospital mortality.

## Discussion

In this single-center, retrospective cohort study, we found that: (1) the rate of IMV was higher in the NIV + HFNO group than the NIV and HFNO groups; (2) the survival rate was lower in the NIV + HFNO group than the HFNO group; and (3) the ICU and hospital lengths of stay were higher in the NIV + HFNO group than the NIV group. After adjustments with the multivariate Cox regression model for comorbidity, chest CT score and SpO_2_/FiO_2_ ratio, the risk of IMV in patients on NIV + HFNO remained high.

The presence of at least one comorbidity, chest CT score and SpO_2_/FiO_2_ ratio was different in our population, because they may contribute to worse clinical outcomes regardless of ventilatory strategies [9, 23]; correction using multivariate regression models is required. Although age [7] and SAPS-3 [8] are well-known risk factors associated with the need for IMV, we did not observe differences in our population between the groups. For instance, SAPS-3 includes several clinical variables and was shown to be a reliable predictor of hospital mortality in patients with COVID-19 admitted to the ICU during the first wave [8] of the pandemic. Our data may reflect the occurrence of P-SILI [24], regardless of chest CT score and SpO_2_/FiO_2_ ratio at admission and the presence of comorbidities, increasing the need for IMV. P-SILI is a life-threatening condition arising from excessive respiratory effort and work of breathing, leading to high transpulmonary pressure and diaphragmatic injury [25]. Positive pressure applied during non-invasive support ventilation can exacerbate P-SILI [26].

We were able to obtain ventilatory variables for 139 of 143 patients in the NIV + HFNO group (3% missing values). We found that 76 patients (54.7%) had an inspiratory airway pressure < 10 cmH_2_O applied during the NIV period, and 63 patients (45.3%) had an inspiratory airway pressure > 10 cmH_2_O. In addition, the highest flow adjusted in the HFNO period in the NIV + HFNO group was 48 ± 10 L/min. Although analyzing the hazard threshold during the NIV and HFNO periods was not our objective in this retrospective study, we recognize that these values may be indicative of a higher likelihood of requiring IMV.

Recent physiologic studies have found that high respiratory effort, as measured by esophageal pressure variation and transpulmonary pressure swings, may be associated with a high rate of failure in non-invasive ventilation. Tonelli et al. [27] showed that an esophageal pressure variation of < 10 cmH_2_O may be linked to a higher rate of orotracheal intubation. Similarly, Grieco et al. [28] found that patients who failed in NIV and required intubation had higher dynamic transpulmonary pressure after 2 h of therapy compared with patients with successful NIV. Our results suggest the importance of identifying patients at increased risk of failure of non-invasive respiratory support due to high respiratory effort [29, 30]. Immediate intubation has been suggested if the PaO_2_/FiO_2_ ratio does not improve and/or PaCO_2_ is < 30 mmHg and/or the respiratory rate is > 28 bpm using accessory muscles for more than 3 h. Colaianni-Alfonso et al. [31] also considered pH < 7.35 as a criterion for intubation, in addition to the other signs. These data suggest that there are no clear guidelines on the signs of failure of NIV.

Despite the variation in exposure time under NIV (from continuous exposure to periods of 2 to 3 times per day), our data show that the total time of therapy (in days) delayed the time to endotracheal intubation, and this can worsen outcomes, mainly when intercalated with HFNO. This reflects that the patient was dependent on some level of positive pressure while not tolerating conventional oxygen therapy between NIV intervals [32]. A previous multicenter retrospective study (COVID–ICU) observed a high risk of IMV leading to a high mortality rate in patients who underwent NIV and/or HFNO [31]. However, a systematic review and meta-analysis of 23 studies and a total of 5354 patients did not show differences in mortality between HFNO and NIV groups [34]. A multicenter, prospective cohort study on patients with COVID-19 showed that HFNO was associated with a reduction in failure of oxygenation without improvement in 90-day mortality, whereas NIV was associated with higher mortality [35]. Although few patients underwent the intercalated use of NIV and HFNO, the authors were able to isolate the effect of HFNO, which was not associated with a reduction in 90-day mortality. Moreover, the NIV group, which was a composite of three conditions, i.e., NIV alone, NIV + HFNO, and NIV + COT, was associated with increased 90-day mortality [33]. In our study, the intercalated use of NIV and HFNO was associated with an increased need for IMV independent of the presence of at least one comorbidity, chest CT score and SpO_2_/FiO_2_ ratio. The rate of IMV and mortality observed in NIV + HFNO group herein was similar to a previous study, in Latin America, using combined noninvasive respiratory support therapies within a similar period of time [36]. The intercalated use of NIV and HFNO was associated with the need for IMV, likely due to the following reasons: (1) the use of NIV alone was also associated with an increased need for IMV in the unadjusted models, suggesting that the hazard condition may be associated with its presence; (2) the periodic application of NIV, which may not have met the patient’s ventilatory support needs; (3) few patients were adapted to HFNO alone, which may have jeopardized the comparisons and may reflect that our population had higher respiratory distress and fatigue on admission; and (4) the decision to implement NIV + HFNO took longer than 24 h in some patients, since delayed application of combined respiratory therapy may be associated with worse outcomes [37]. When intense respiratory effort was present, the clinical decision favored implementation of NIV. The intercalated use of NIV and HFNO makes decision-making harder, because it may prolong the time under non-invasive support and give the false impression that the patients do not need intubation and IMV. However, objective indexes and criteria should be applied to help identify the ideal time to stop non-invasive support and consider IMV. All these results raise the question whether these findings apply only to patients with COVID-19 or may be similar in other patients. More studies are needed to investigate the intercalated use of non-invasive support in cases of non-COVID 19-hypoxemic acute respiratory failure.

Our study has some limitations that should be considered. First, due to the exploratory nature of the research, data collection was based on medical records, which may have limited the analyses. Nevertheless, our study may reflect real life with a large sample size. Second, because this is a retrospective, observational, single-center study, causal inference is not possible. Third, data collection took place mainly during a period with no vaccines; the first vaccine in Brazil was given in January 2021. Our data may be particularly relevant in undeveloped countries where the rate of routine immunization for adults is low. Fourth, our sample included a relatively small number of patients under HFNO alone. Fifth, this study encompasses a single tertiary hospital, not a multicenter public hospital.

## Conclusion

In patients with COVID-19, NIV alternating with HFNO was associated with a higher rate of IMV independent of the presence of comorbidities and chest CT score. However, due to the study design, residual confounding due to the severity of the disease is likely.

### Supplementary Information

**Additional file 1: Figure S1. **Description of interventions. COT, conventional oxygen therapy; HFNO, high-flow nasal oxygen; NIV, non-invasive ventilation; RT–PCR, reverse transcription–polymerase chain reaction.

**Additional file 2: Table S1.** Characteristics of patients with COVID-19 who underwent conventional oxygen therapy in the hospital between March 2020 and July 2021.

**Additional file 3: Table S2.** Ventilatory variables during NIV, HFNO, and NIV+HFNO.

**Additional file 4: Table S3.** Causes of failure of non-invasive therapy in the NIV, HFNO, and NIV+HFNO groups among patients who needed invasive mechanical ventilation.

## Data Availability

The data sets used and/or analyzed during the current study are available from the corresponding author on reasonable request.

## Abbreviations

BMI: Body mass index
CI: Confidence interval
COT: Conventional oxygen therapy
CT: Computed tomography
HFNO: High-flow nasal oxygen
HR: Hazard ratio
ICU: Intensive care unit
IMV: Invasive mechanical ventilation
NIV: Non-invasive ventilation
P-SILI: Patient self-inflicted lung injury
SAPS-3: Simplified Acute Physiology Score-3
