# Supplementary figures and images for: Comparison between high-flow nasal oxygen (HFNO) alternated with non-invasive ventilation (NIV) and HFNO and NIV alone in patients with COVID-19: a retrospective cohort study
Source: Eur J Med Res. 2024 Apr 22;29:248. doi: 10.1186/s40001-024-01826-3 (PMC11036698; doi:10.1186/s40001-024-01826-3)

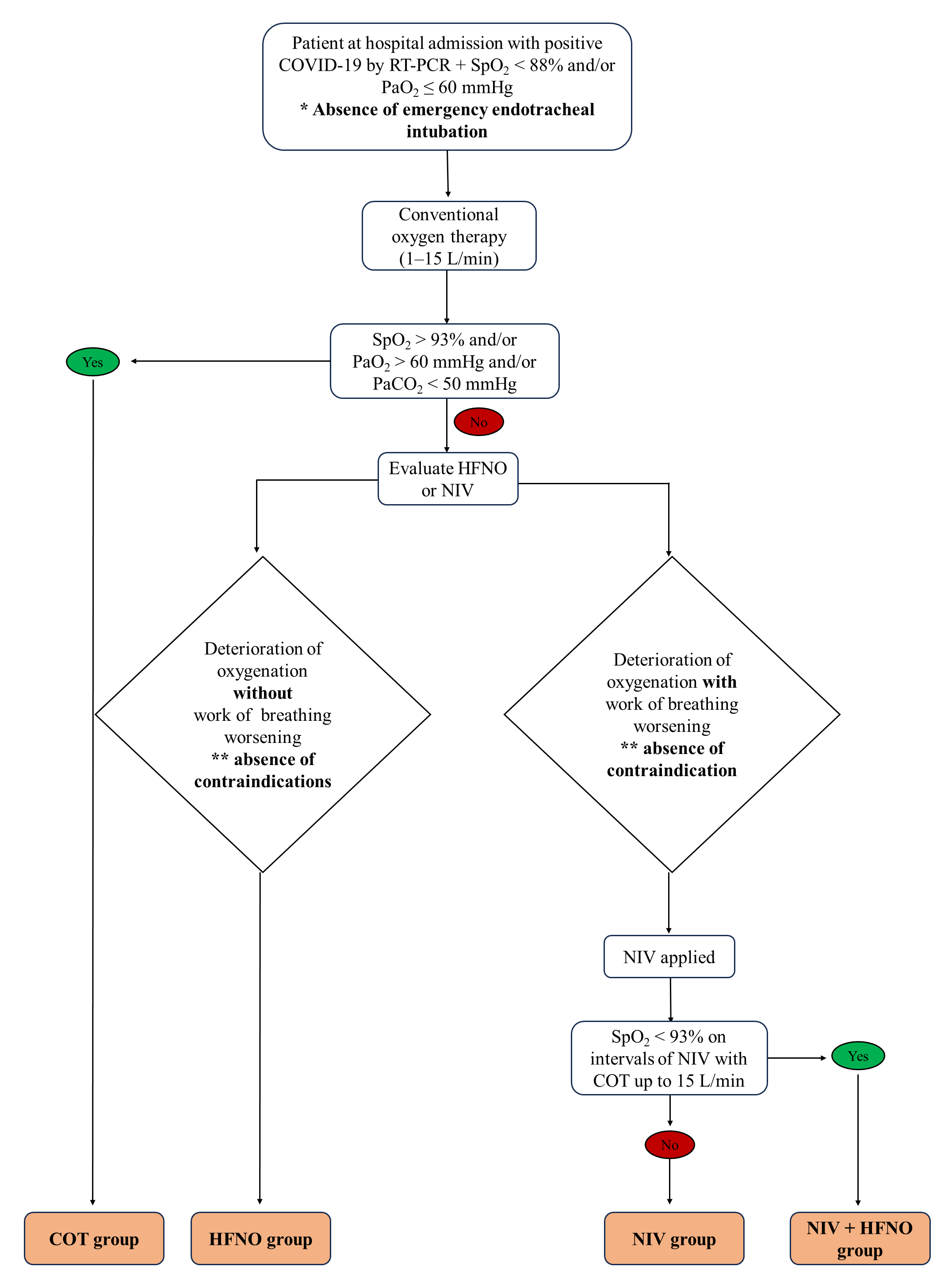

Supplement: Supplementary file 1 — Additional file 1: Figure S1. Description of interventions. COT, conventional oxygen therapy; HFNO, high-flow nasal oxygen; NIV, non-invasive ventilation; RT–PCR, reverse transcription–polymerase chain reaction. [file 40001_2024_1826_MOESM1_ESM.tif]
