# Supplementary material for: Comparison between high-flow nasal oxygen (HFNO) alternated with non-invasive ventilation (NIV) and HFNO and NIV alone in patients with COVID-19: a retrospective cohort study
Source: Eur J Med Res. 2024 Apr 22;29:248. doi: 10.1186/s40001-024-01826-3 (PMC11036698; doi:10.1186/s40001-024-01826-3)
Supplement: Supplementary file 2 — Additional file 2: Table S1. Characteristics of patients with COVID-19 who underwent conventional oxygen therapy in the hospital between March 2020 and July 2021. [file 40001_2024_1826_MOESM2_ESM.docx]

**Additional File 2, Table S1** Characteristics of patients with COVID-19 who underwent conventional oxygen therapy in the hospital between March 2020 and July 2021

|  | **All patients** | **COT** |
| --- | --- | --- |
| Absolute and relative frequencies, *n* (%) | 958 | 692 (72.2) |
| Age (years), median (IQR) | 59 (47–72) | 58 (46–70) |
| Sex, *n* (%) |  |  |
| Male | 609 (63.6) | 424 (61.3) |
| Female | 349 (36.4) | 268 (38.7) |
| Comorbidities, *n* (%) |  |  |
| COPD | 33 (3.4) | 20 (2.9) |
| Asthma | 41 (4.3) | 26 (3.8) |
| Cerebrovascular disease | 11 (1.1) | 8 (1.2) |
| Heart failure | 38 (4.0) | 27 (3.9) |
| Hypertension | 466 (48.6) | 299 (43.2) |
| Diabetes mellitus | 250 (26.1) | 164 (23.7) |
| Kidney failure | 42 (4.4) | 21 (3.0) |
| Immunosuppression | 2 (0.2) | 1 (0.1) |
| Other comorbidities | 547 (57.0) | 377 (54.5) |
| BMI, *n* (%) |  |  |
| <29.9 kg/m^2^ | 433 (45.1) | 320 (64.3) |
| ≥30.0 kg/m^2^ | 269 (28.0) | 178 (35.7) |
| SAPS-3 score, median (IQR)^a^ | 47 (42–53) | 46 (39–52) |
| **Clinical parameters** |  |  |
| Time from symptom onset to hospital admission *n* (%) |  |  |
| 0–10 days | 494 (51.5) | 362 (89.2) |
| 11–20 days | 44 (4.6) | 34 (8.4) |
| 21–30 days | 9 (0.9) | 5 (1.2) |
| >30 days | 6 (0.6) | 5 (1.2) |
| Chest CT score, *n* (%) |  |  |
| 0%–25% | 89 (9.3) | 74 (12.3) |
| 26%–50% | 649 (67.7) | 484 (80.4) |
| >50% | 100 (10.4) | 40 (6.6) |
| Undetermined | 7 (0.7) | 4 (0.7) |
| Normal CT scan | 114 (11.9) |  |
| Concomitant medications, *n* (%) |  |  |
| Azithromycin | 425 (44.3) | 285 (41.2) |
| Amoxicillin/clavulanic acid | 212 (22.1) | 174 (25.1)† |
| Dexamethasone | 645 (67.3) | 408 (59.0) |
| Kidney replacement therapy for acute kidney injury (dialysis) | 104 (10.8) | 33 (6.0) |
| ICU length of stay, (days) | 7 (3–15) | 4 (2–8) |
| Hospital length of stay, (days) | 8 (5–15) | 6 (4–10) |

The descriptive analysis of the data is presented as absolute frequencies (*n*) and percentages according to the group except where indicated otherwise. COT, conventional oxygen therapy; IQR, interquartile range; COPD, chronic obstructive pulmonary disease; BMI, body mass index (calculated as weight in kilograms divided by height in meters squared); SAPS, Simplified Acute Physiology Score; CT, computed tomography.

^a^SAPS-3 estimates the probability of mortality for patients in the intensive care unit (ICU) on admission using patient characteristics, indication for ICU admission, and physiologic derangement on ICU admission.
