# Supplementary material for: Comparison between high-flow nasal oxygen (HFNO) alternated with non-invasive ventilation (NIV) and HFNO and NIV alone in patients with COVID-19: a retrospective cohort study
Source: Eur J Med Res. 2024 Apr 22;29:248. doi: 10.1186/s40001-024-01826-3 (PMC11036698; doi:10.1186/s40001-024-01826-3)
Supplement: Supplementary file 3 — Additional file 3: Table S2. Ventilatory variables during NIV, HFNO, and NIV+HFNO. [file 40001_2024_1826_MOESM3_ESM.docx]

**Additional File 3, Table S2** Ventilatory variables during HFNO, NIV and NIV+HFNO

|  | NIV (*n*=92) | HFNO (*n*=31) | NIV+HFNO (*n*=143) | *p* value |
| --- | --- | --- | --- | --- |
| Duration of ventilatory therapies (days), median (Q1–Q3) | 6 (4–8) | 5 (4–8) | 8 (5–11)*# | <0.001 |
| Inspiratory airway pressure, *n* (%) |  |  |  |  |
| <8 cmH_2_O | 5 (6.5) |  | 8 (5.8) | 0.953 |
| 8–10 cmH_2_O | 40 (51.9) |  | 68 (48.9) |  |
| 11–13 cmH_2_O | 21 (27.3) |  | 40 (28.8) |  |
| >14 cmH_2_O | 11 (14.3) |  | 23 (16.5) |  |
| Expiratory airway pressure, *n* (%) |  |  |  |  |
| <8 cmH_2_O | 8 (10.3) |  | 6 (4.3) | 0.332 |
| 8–9 cmH_2_O | 41 (52.6) |  | 71 (51.1) |  |
| 10–11 cmH_2_O | 27 (34.6) |  | 58 (41.7) |  |
| >11 cmH_2_O | 2 (2.6) |  | 4 (2.9) |  |
| Oxygen therapy used between ventilatory therapies, *n* (%) |  |  |  |  |
| ≤1 L/min | 18 (21.2) | 0 | 29 (26.8) | 0.003 |
| 2–5 L/min | 28 (32.9)†# | 1 (9.1) | 15 (13.9) |  |
| 6–9 L/min | 39 (45.9) | 10 (90.9)*† | 64 (59.3) |  |
| Highest oxygen flow during HFNO (L/min), mean±SD | – | 42±8 | 48±10 |  |
| Lowest oxygen flow during HFNO (L/min), mean±SD | – | 34±9 | 38±11 |  |

Bonferroni multiple comparison tests were done for proportions or continuous variables, as appropriate. NIV, non-invasive ventilation; HFNO, high-flow nasal oxygen. *versus the NIV group; #versus the HFNO group; † versus the NIV+HFNO group.
