# Supplementary material for: Comparison between high-flow nasal oxygen (HFNO) alternated with non-invasive ventilation (NIV) and HFNO and NIV alone in patients with COVID-19: a retrospective cohort study
Source: Eur J Med Res. 2024 Apr 22;29:248. doi: 10.1186/s40001-024-01826-3 (PMC11036698; doi:10.1186/s40001-024-01826-3)
Supplement: Supplementary file 4 — Additional file 4: Table S3. Causes of failure of non-invasive therapy in the NIV, HFNO, and NIV+HFNO groups among patients who needed invasive mechanical ventilation. [file 40001_2024_1826_MOESM4_ESM.docx]

**Additional File 4, Table S3** Causes of failure after NIV, HFNO, and NIV+HFNO strategies among patients who needed invasive mechanical ventilation

|  | **Patients who needed IMV (*n*=146)** | **NIV (*n*=24)** | **HFNO (*n*=7)** | **NIV+HFNO (*n*=67)** | ***p* value** |
| --- | --- | --- | --- | --- | --- |
| Worsening of the work of breathing, *n* (%) | 74 (50.7) | 9 (37.5) | 5 (71.4) | 37 (55.2) | 0.186 |
| Clinical decision by medical staff, *n* (%) | 28 (19.2) | 6 (25) | 0 (0) | 18 (26.9) | 0.290 |
| Hypoxemia and/or acidosis, *n* (%) | 16 (11) | 1 (4.2) | 1 (14.3) | 8 (11.9) | 0.521 |
| Low level of consciousness, *n* (%) | 12 (8.2) | 5 (20.8) † | 1 (14.3)† | 0 (0) | <0.001 |
| Cardiopulmonary resuscitation event, *n* (%) | 2 (1.4) | 0 (0) | 0 (0) | 1 (1.5) | 0.792 |
| Intolerance to therapy and/or face mask, *n* (%) | 1 (0.7) | 0 (0) | 0 (0) | 1 (1.5) | 0.792 |
| Other, *n* (%) | 8 (5.5) | 0 (0) | 0 (0) | 2 (3.0) | 0.624 |
| Cause missing, *n* (%) | 5 (3.4) | 3 (12.5) | 0 (0) | 0 (0) | 0.008 |

Bonferroni multiple comparison tests were done for proportions or continuous variables, as appropriate. IMV, invasive mechanical ventilation; NIV, non-invasive ventilation; HFNO, high-flow nasal oxygen. †Versus the NIV+HFNO group.
